# A dual role for ERK-1/2 in the regulation of plasmin activity and cell migration in metastatic NSCLC-H1299 cells

**DOI:** 10.1007/s00204-023-03600-6

**Published:** 2023-09-15

**Authors:** Sarah Zeitlmayr, Ditila Cami, Belinda Selmani, Thomas Gudermann, Andreas Breit

**Affiliations:** Walther Straub Institute of Pharmacology and Toxicology, Medical Faculty, LMU Munich, Goethestrasse 33, 80336 Munich, Germany

**Keywords:** Lung cancer, TGF-β, Plasmin, ERK-1/2, H1299, A549

## Abstract

Occupational and environmental exposure of various toxins or cigarette smoke causes non-small cell lung carcinoma (NSCLC); a devastating disease with a very low survival rate after metastasis. Increased activity of plasmin is a hallmark in NSCLC metastasis. It is accepted that metastatic cells exhibit higher plasmin activity than cells from primary tumors. Mechanisms behind this elevation, however, are barely understood. We compared plasmin activity and cell migration of A549 cells derived from a primary lung tumor with metastatic H1299 lung cells isolated from lymph nodes. Surprisingly, we found higher plasmin activity and migration for A549 cells. mRNA levels of the plasminogen activator inhibitor-1 (PAI-1) were higher in H1299 cells and activity of extracellular-regulated kinases-1/2 (ERK-1/2) was increased. An inhibitor of ERK-1/2 decreased PAI-1 mRNA levels and increased plasmin activity or cell migration in H1299 cells. Transforming growth factor-β (TGF-β) decreased plasmin activity and migration in A549 cells but enhanced both in H1299 cells. The cytokine massively increased PAI-1 and decreased urokinase plasminogen activator (uPA) levels in A549 cells but strongly induced uPA and only weakly PAI- 1 expression in H1299 cells. Consequently, TGF-β enhanced plasmin activity and cell migration in H1299. Additionally, TGF-β activated ERK-1/2 stronger in H1299 than in A549 cells. Accordingly, an ERK-1/2 inhibitor completely reversed the effects of TGF-β on uPA expression, plasmin activity and migration in H1299 cells. Hence, we provide first data indicating TGF-β-promoted increased plasmin activity and suggest that blocking TGF-β-promoted ERK-1/2 activity might be a straightforward approach to inhibit NSCLC metastasis.

## Introduction

Non-small cell lung carcinoma (NSCLC) occur with an 85% incidence among lung cancers and exhibit a five-year survival rate of less than 5% after metastasis (Molina et al. 2008; Torre et al. 2016). First and secondhand smoking as well as occupational exposure to toxins such as asbestos, arsenic, nickel chromium or tar and soot increase the risk of NSCLC (Klebe et al. 2019). Further, sustained environmental exposure in homes and offices to radon can cause NSCLC (Bochicchio et al. 2005; Ratnasinghe et al. 2000). Thus, around 1.8 million people per year lose their lives due to lung cancer worldwide. Furthermore, it is under debate that SARS-CoV-2 might induce oncogenic mechanisms associated with lung cancer development, suggesting that the incidence of NSCLC might even further increase in the years to come (Khiali et al. 2022).

Degradation of basement membrane (BM) and remodeling of extracellular matrix (ECM) are key factors in cancer cell migration and ultimately contribute to the development and progression of metastasis (Duffy 1992; Ludwig 2005). Consequently, knowledge about the molecular and cellular mechanism regulating BM and ECM could help to identify novel therapeutic targets as well as to develop new strategies against lung cancer.

The fibrinolytic system is a major regulator of BM or ECM (Smit et al. 1999; Wong et al. 1992). It is composed of the proteolytic enzyme plasmin, its precursor plasminogen (Plg) and the urokinase plasminogen activator (uPA). Urokinase activity, and thus plasmin activity, is counterbalanced by the plasminogen activator inhibitor-1 (PAI-1). Once activated by uPA, plasmin activity is also diminished by α_2_-antiplasmin (Castellino and Ploplis 2005; Law et al. 2013). Thus, plasmin activity results from the expression of multiple proteins, interacting in an intertwining network.

Due to its key role in the regulation of BM and ECM, enhanced plasmin activity directly contributes to the development and progression of metastasis (Szende et al. 2002; Tan et al. 2006). Hence, components of the fibrinolytic system such as uPA have been considered as anti-metastatic drug targets (El Salamouni et al. 2022). In contrast, a paradoxical role in cancer progression has been described for PAI-1. Despite its inhibitory action on plasmin activity, in vitro experiments demonstrated that PAI-1 facilitates tumor cell migration (Kubala and DeClerck 2019). However, considering that plasmin activity is the actual parameter by which PAI-1 and uPA effect cell migration, expression ratios of PAI-1 versus uPA might be more decisive than absolute expression levels. Of note, tumor cell migration data at different PAI-1/uPA expression ratios have rarely been directly correlated with actual measurements of plasmin activity or cell migration. Thus, although it is widely accepted that migrating metastatic cancer cells exhibit higher plasmin activity than cells from primary tumors, mechanisms or conditions responsible for altered PAI-1/uPA expression ratios and thus elevated plasmin activity are rather unknown (Bharadwaj et al. 2021).

TGF-β activates transmembrane receptors of the serine/threonine kinase family and is a major regulator of both plasmin activity and cell migration. Its inhibitory actions on plasmin activity are attributed to enhanced PAI-1 levels via activation of SMAD transcription factors (Dong et al. 2001; Macias et al. 2015). Despite its negative effects on plasmin activity via PAI-1, TGF-β has been reported to enhance tumor cell migration independently from SMADs, by remodeling cytoskeleton via monomeric G proteins of the rho/rac family (Tsai et al. 2014; Ungefroren et al. 2018). Further, TGF-β also activates the RAF/MEK/ERK-1/2 pathway in NSCLC in a SMAD independent manner (Kong et al. 2014; Yu et al. 2020). However, a connection of this pathway to plasmin activity or cell migration has not yet been observed. Of note, because TGF-β activates pro- and anti-migrative signaling pathways, its actual effects on cell migration might be situationally different and not fully comprehended. Along with this notion, TGF-β plays a paradoxical role in cancer: it acts as a tumor suppressor in early-stage tumors, but promotes tumor progression and metastasis in later stages (Zhang et al. 2014).

NSCLC are classified into lung adenocarcinoma, squamous or large cell carcinoma. Here, we took advantage of two NSCLC cell lines with different lineage and metastatic potential: A549 cells derived from a primary lung adenocarcinoma and H1299 large cell lung carcinoma cells isolated from lymph nodes (Giaccone et al. 1992; Giard et al. 1973; Phelps et al. 1996; Wu et al. 2019). We compared plasmin activity, mRNA expression of the *SERPINE-1* (PAI-1), *PLAU* (uPA)*, c-FOS, c-JUN* and *RAF-1* gene, cell migration and death. We found that under basal conditions H1299 cells exhibited less plasmin activity and cell migration most likely due to enhanced ERK-1/2 activity and PAI-1 expression. After challenging H1299 cells with TGF-β, higher ERK-1/2 activity, uPA expression, plasmin activity and cell migration compared to A549 cells was observed. The distinctions between both cell lines were removed by an ERK-1/2 inhibitor. In cell proliferation assays, TGF-β acted anti-proliferative on A549 but not on H1299 cells. Overall, we conclude that the metastatic-like phenotype of H1299 cells depends on the state of ERK-1/2 activity and on the presence of TGF-β. Our data highlight the ERK-1/2 pathway as a therapeutic target against lung cancer metastasis and partly resolve the TGF-β paradox.

## Materials and methods

### Chemicals and antibodies

Verteporfin, human transforming growth factor (TGF-β1; T7039), plasminogen and D-Val-Leu-Lys-7-amido-4-methylcoumarin were from SigmaAldrich. OXA-06, PD-184352, SR-11302 were from Tocris. For protein detection specific antibodies against PAI-1 (Abcam Cat# ab66705, RRID:AB_1310540), fibronectin (abcam, ab2413), p-ERK-1/2 (Santa Cruz, E4, sc-7383), SDHA (abcam, ab14715) and histone H3 (abcam, ab1791) were used.

### Cell culture

A549 cells (CCL-185) were obtained from ATCC. H1299 cells (NCI-DTP Cat# NCIH1299, RRID:CVCL_0060) were kindly provided by Dr. Georgios Stathopoulos, Comprehensive Pneumology Center, Munich, Germany. SK-Lu-1 (Cat # CB-93120835), BEAS-2B (Cat # 95102433) and 16-HBE-14o- (Cat # SCC-150) were from SigmaAldrich. All cell lines were cultured in RPMI 1640 (Gibco Roswell Park Memorial Institute medium 1640) medium containing 10% fetal bovine serum and 100U/ml penicillin and streptomycin form Gibco, Waltham (15070-063).

### Plasmin activity

**Fig. 1:**
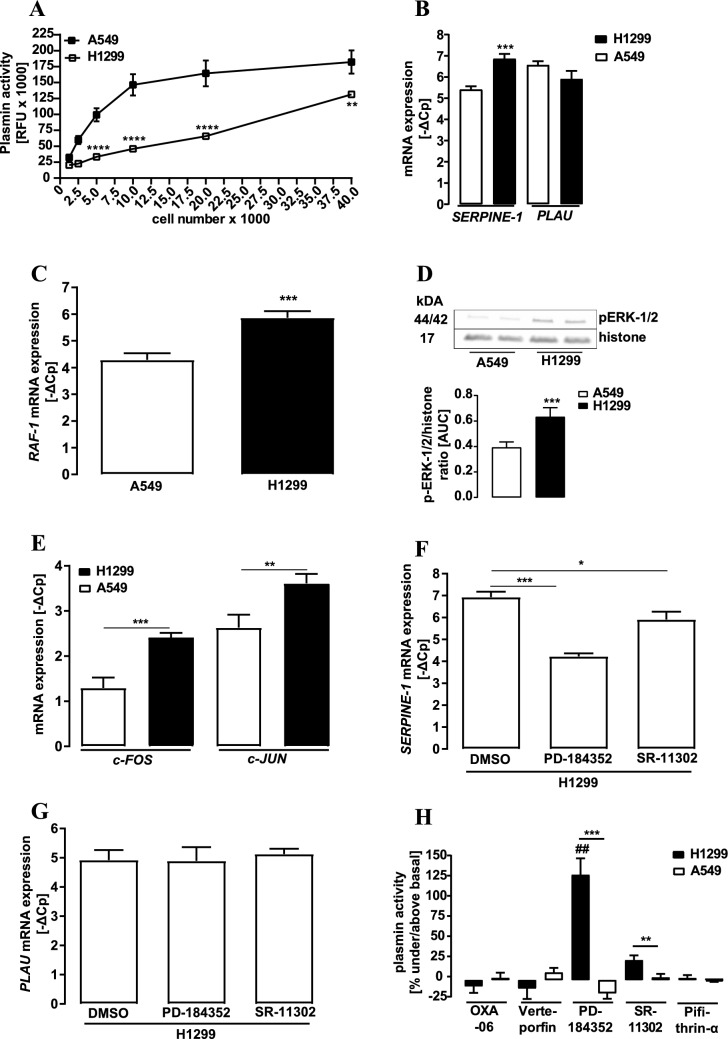
Detection of plasmin activity, mRNA levels and ERK-1/2 phosphorylation in NSCLC cells. **a** D-Val-Leu-Lys-AMC (50 µM) was incubated with the supernatant of NSCLC cells and fluorescence measured. **b** Basal mRNA levels of *SERPINE-1* and *PLAU* and in **c** of *RAF-1* were detected by qRT-PCR. **d** Phosphorylation of ERK-1/2 was detected by western-blotting using a p-ERK-1/2 specific antibody. Detection of histone-3 served as a loading control. **e** *c-FOS* and *c-JUN* mRNA levels were detected by qRT-PCR. **f** *SERPINE-1* and **g** *PLAU* mRNA levels were detected in H1299 cells by qRT-PCR after stimulation of the cells with PD-184352 (10 µM), SR11302 (1 µM) or DMSO (0.1%) for 24 h. **h** Plasmin activity was measured after incubation of NSCLC cells with OXA-6 (5 µM), verteprofin (1 µM), PD-184352 (10 µM), pifithrin-α (30 µM) or SR-11302 (1 µM) or the corresponding carrier control for 24 h. Plasmin activity detected for the control was set to 100%. Statistical analysis was performed using two‐way ANOVA (**a**, **b**, **e**, **f**, **g**, **h**) followed by Tukey´s post-test or one- and two-sample t-test (**c**, **d**, **h**). Asterisks indicate in **a**–**e** significant differences between the cell lines, in **f** and **h** between inhibitor and control. **h** hash signs indicate significant differences to zero

D-Val-Leu-Lys-7-amido-4-methylcoumarin (D-Val-Leu-Lys-AMC) was used as a plasmin substrate (Gyzander and Teger-Nilsson 1980; Kato et al. 1980; Li et al. 2018; Schuliga et al. 2011; Wu et al. 2019; Zeitlmayr et al. 2022). D-Val-Leu-Lys-AMC is a selective fluorogenic substrate for plasmin and enzymatic activity is quantified by release of the free AMC fluorophore, which is excited at 360–380 nm and emits light at 440–460 nm. In Fig. 1A, indicated number of cells were seeded per cavity of a 48-well plate 24 h before the experiment. After 24 h, 10 µl of the supernatant was transferred to a 96-well plate and added to 90 µl Tris/HCl (20 mM, pH 7.4) containing 55 µM of D-Val-Leu-Lys-AMC. After 3 h incubation at 37 °C, fluorescence was measured using a FLUOstar® Omega plate reader. In all other experiments ~ 5000 cells were seeded in a cavity of a 96-well plate 24 h before the experiment and then proceeded as described above. In order to normalize fluorescence signals to the total protein amount, a second 96-well plate was prepared, treated equally and protein amount monitored by sulforhodamine B (SRB) colorimetric assay (Vichai and Kirtikara 2006). SRB signals were calibrated to the cell number. Plasmin activity was determined as the ratio of the RLU values of the D-Val-Leu-Lys-AMC detection and the OD values of the SRB measurement.

### Protein detection by western-blotting

Cells were seeded on 6-well plates (~ 200,000/well), cultured for one day and stimulated for the indicated periods of time. In order to detect expression of secreted proteins, supernatants were transferred to fresh tubes and lysed with Laemmli buffer (fourfold). The corresponding cell fraction was lysed by directly adding Laemmli buffer (onefold) to the 6-well plates. Lysates were subjected to SDS-PAGE (10%) and proteins transferred to nitrocellulose (Amersham Protran™ 0.45 µm, #10600002) by western-blotting. After adding the primary antibody over night at 4 °C, blots were washed and incubated with the corresponding HRP-conjugated secondary antibody (anti-rabbit 1:4,000, anti-mouse 1:2,000) for 1 h at RT. After intensive washing, immune reactivity was detected by monitoring the ECL dependent light emission with a chemiluminescence detection system (Peqlab, Germany). Resulting signals were quantified by densitometry (ImageJ, RRID: SCR_003070) and ratios of the protein of interest and the loading control calculated.

### Firefly luciferase reporter gene assay

In order to monitor SMAD activation, the pCAGA-luc reporter construct containing the SMAD-3/4 sensitive part of the human *SERPINE-1* promotor was used (Dennler et al. 1998). Plasmids were transfected into cells using TurboFectTM reagent from Thermofisher according to the manufacturer´s protocol. Briefly, ~ 70,000 cells were seeded on 12-well plates and cultivated for 24 h. For each well, the equivalent volume to 250 ng Plasmid in 50 μl serum free medium was mixed with the double volume of TurboFect™ reagent in 50 μl serum free medium and incubated for 30 min at RT. 100 μl of this mixture was added to each well, cells were incubated for 24 h and hereafter stimulated for the desired time periods. After stimulation, cells were lysed in 200 µl of lysis buffer (25 mM Tris/HCl pH 7.4, 4 mM EGTA, 8 mM MgCl2, 1 mM DTT and 1% Triton-X-100) and a volume of 180 µl transferred to white-bottomed, 96-well plates. Luciferase activity was measured after automatically injecting a luciferase substrate (40 µl) from Promega (E1500) using a FLUOstar® Omega plate reader.

### mRNA detection by qRT-PCR

~ 150,000 cells were seeded per cavity of a 6-well plate. After 24 h, cells were stimulated or not for the time indicated and stimulation terminated by rapid cooling on ice. Total RNA was isolated using the Trizol® reagent (Invitrogen, Darmstadt, Germany) according to the manufacturer´s instructions. First strand synthesis was carried out with oligo(dT)_18_ primer using 1 µg of total RNA and the RevertAid™ H Minus First Strand cDNA Synthesis Kit (Fermentas, Sankt-Leon Roth, Germany). qRT-PCR was done using the LightCycler® 480 SybrGreen I Master Mix kappa (Roche, Mannheim, Germany). Exon-spanning primer pairs were used at a final concentration of 1 µM each. Final assay volume was 20 µl and first strand synthesis reaction was diluted 1:20 or 1:50. A LightCycler® 480 II (Roche) was used with the following conditions: initial denaturation for 2 min at 94 °C, 55 cycles of 94 °C for 10 s, 55 °C for 10 s and 72 °C for 10 s. Primer design was performed using the ProbeFinder (RRID:SCR_014490) provided on the website of Roche Life Science. Crossing points (Cp) were determined by the software supplied with the LightCycler® 480 and data analysed by the ΔCp or ΔΔCp method (2^−((gene–*ACTB*)^_TGF-β_^−(gene–*ACTB*))^_basal_). Primer sequences were as follows: *SERPINE1*-forward: 5ʹ-AAGGCACCTCTGAGAACTTCA-3ʹ, *SERPINE1*-reverse: 5ʹ-CCCAGGACTAGGCAGGTG-3ʹ, *ACTB*-forward: 5ʹ-CTAAGGCCAACCGTGAAAAG-3ʹ, *ACTB*-reverse: 5ʹ-ACCAGAGGCATACA-GGGACA-3ʹ, *PLAU*-forward: 5ʹ-AGTGTCAGCAGCCCCACT-3ʹ, *PLAU*-reverse: 5ʹ-CCCCCTGA-GTCTCCCTGG-3ʹ, *c-FOS*-reverse: 5ʹ-AGTTGGTCTGTCTCCG-CTTG-3ʹ, c-*FOS*-forward: 5ʹ-GG-GGCAAGGTGGAACAGTTA-3ʹ, c-*JUN*-forward: 5ʹ-CC-AACTCATGCTAACGCAGC-3ʹ, *c*-*JUN*-reverse: 5ʹ-TCTCTCCGTCGCAACTTGTC-3ʹ, *RAF-1*-forward: 5ʹ-GGGGCTTGGAAGACGATC-AG-3ʹ, *RAF-1*-reverse: 5ʹ-ACACGGA-TAGTGTGCTGTC-3ʹ.

### Phospho-SMAD-2 enzyme-linked immunosorbent assay (ELISA)

SMAD-2 phosphorylation at Serine 465/467 was determined using the PathScan® Phospho-SMAD-2 Sandwich ELISA Kit from CellSignal (7870c). 50,000 cells were seeded onto 24-well plates, kept overnight and stimulated for the desired times. ELISA was conducted according to the protocol provided by the manufacturer. Absorbance at 450 nm was detected with the FLUOstar Omega microplate reader and displayed as (OD).

### Migration assay

Migratory properties of cells were monitored by a Boyden chamber migration assay (Boyden 1962; Chen 2005). A membrane with 5 μM pore size was used to identify cells that are able to change their cytoskeleton to actively migrate through these pores. Migratory cells were then dyed, dye was dissolved and absorption was measured to quantify migratory cells. Dying of the cells was performed with sulforhodamine B (SRB), which determines total protein amount. ~ 250,000 cells were placed in cell culture inserts in 100 μl growth medium and inserts were placed in 24-well plates containing 500 μl growth medium (Vichai and Kirtikara 2006). A part of the inserts was submerged in the medium from the lower chamber and incubated for 24 h. Medium in upper and lower chambers was then replaced with medium containing the desired reagent. Cell migration was monitored by using three distinct protocols: (I) 0.5% FBS in both chambers, (II) 0.5% in the upper and 10% FBS in the lower chamber and (III) 10% FBS in both chambers. Effects of TGF-β were measured by adding the reagent to the upper chamber. After 24 h stimulation, medium within the inserts was aspirated and inserts were placed in a new well containing 400 μl SRB dye. After 1 h, inserts were dipped in 1% acetic acid to remove unbound dye and cells on top of the membrane were removed thoroughly with cotton buds. Migrated cells remained dyed on the bottom of the membrane. The insert was then placed in a new well containing 200 μl TRIS-base (pH 10.5) and incubated on a shaker for 20 min until dye was completely dissolved. Samples were transferred to a 96-well plate and absorbance at 510 nm was measured with the FLUOstar Omega microplate reader and displayed as OD.

### Cytotoxicity assay

~ 5000 cells were seeded on 96-well plates. After 24 h, cells were stimulated or not and further cultured for 24 or 120 h, respectively. Total protein amount were determined using SRB (Vichai and Kirtikara 2006).

### Quantification and statistical analysis

Values represent the mean ± SEM of five to ten independent experiments. Statistical analysis was performed using one- or two-sample student’s t-test, one‐way or two-way ANOVA followed by Tukey´s post-test using the GraphPad prism software 9.1 (RRID:SCR_002798). Shapiro–Wilk tests were performed in order to ensure normal distribution of the data sets. One symbol indicates a *p*‐value of ≤ 0.05, two of ≤ 0.01 and three of ≤ 0.001.

## Results

### Increased ERK-1/2 activity elevates AP-1 and thus PAI-1 expression in metastatic H1299 NSCLC cells leading to reduced plasmin activity

Despite the importance of the plasmin system for lung cancer metastasis, plasmin activity has not been systematically investigated in distinct NSCLC cell lines. Hence, we used D-Val-Leu-Lys-AMC as an established plasmin substrate (Gyzander and Teger-Nilsson 1980; Kato et al. 1980; Li et al. 2018; Wu et al. 2019; Zeitlmayr et al. 2022) and compared plasmin activity of A549 adenocarcinoma cells derived from a primary lung tumor with metastatic H1299 large cell lung carcinoma cells. Surprisingly, over a wide range of cell numbers, H1299 cells exhibited significantly less plasmin activity than A549 cells (Fig. 1A). In order to obtain first insights into this intriguing finding, we analyzed mRNA levels of the SERPINE-1 and the PLAU gene by qRT-PCR in both cell lines (Fig. 1B). SERPINE-1 mRNA levels were significantly higher in H1299 cells, suggesting that enhanced PAI-1 protein levels account for lower plasmin activity in H1299 cells. The promoter of the SERPINE-1 gene contains binding sites for SMAD-3/4 and AP-1 (Dennler et al. 1998). The AP-1 complex consists of c-JUN and c-FOS transcription factors, which are induced by ERK-1/2 activity (Yang et al. 1997). Previous data suggested that expression of the ERK-1/2 kinase RAF-1 is enhanced in H1299 compared to A549 cells (Qiu et al. 2019). In line with these previous data, we detected higher RAF-1 mRNA levels in H1299 cells (Fig. 1C). Interestingly, higher RAF-1 levels are indeed translated into increased ERK-1/2 phosphorylation in H1299 cells (Fig. 1D), suggesting that this pathway leads to increased PAI-1 expression via AP-1. In line with this hypothesis, c-FOS and c-JUN mRNA levels were both significantly increased in H1299 cells (Fig. 1E). In order to analyze a link between enhanced ERK-1/2 activity and c-FOS/c-JUN expression on one side and enhanced SERPINE-1 mRNA levels on the other, we tested effects of the ERK-1/2 inhibitor PD-184352, and of the AP-1 inhibitor SR-11302 on SERPINE-1 and PLAU expression in H1299 cells (Fig. 1F, G). Both inhibitors significantly decreased SERPINE-1 but not PLAU mRNA levels. Thus, our data suggest that increased RAF-1 levels lead to enhanced ERK-1/2 activity, AP-1 and PAI-1 expression in H1299 cells and thus subsequently to reduced plasmin activity. To finally validate this hypothesis, we next analyzed effects of distinct inhibitors on plasmin activity in NSCLC cells. OXA-6, pifithrin-α and verteporfin, inhibitors of rho-dependent kinases, p53 or YAP transcription factors, were without any effect in both cell lines. However, PD-184352 and SR-11302 significantly enhanced plasmin activity in H1299 but not in A549 cells (Fig. 1H), confirming that enhanced ERK-1/2 activity and AP-1 expression account for reduced plasmin activity in H1299 cells.

### Enhanced TGF-β-induced ERK-1/2 activity increases uPA expression and thus elevates plasmin activity and migration of metastatic H1299 cells

**Fig. 2:**
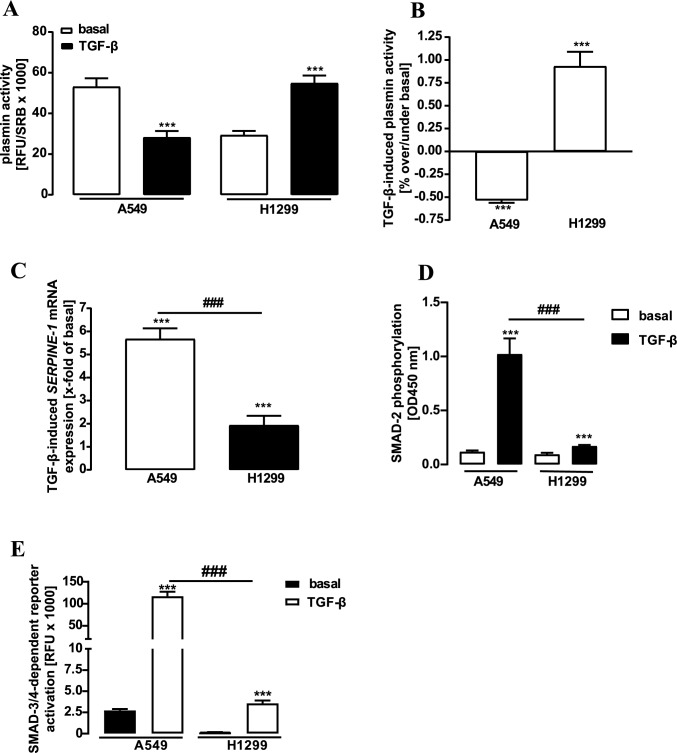
Detection of plasmin activity, mRNA levels, SMAD-2 phosphorylation and SMAD-3/4-dependent reporter activity in NSCLC cells. **a** D-Val-Leu-Lys-AMC (50 µM) was incubated with the supernatant of unstimulated or TGF-β treated (2 ng/ml for 24 h) cells and fluorescence measured. **b** The same data shown in **a** were normalized by setting basal to 100%. **c** TGF-β-induced (2 ng/ml for 24 h) *SERPINE-1* mRNA levels of H1299 cells were detected by qRT-PCR. **d** TGF-β-induced (2 ng/ml for 24 h) p-SMAD-2 phosphorylation in NSCLC cells was detected. **e** NSCLC cells were transfected with the SMAD-3/4-dependent reporter plasmid. After 24 h, cells were stimulated with TGF-β (2 ng/ml) for 24 h and then luciferase activity determined. Statistical analysis was performed using two‐way ANOVA **a** followed by Tukey´s post-test or one- and two-sample t-test (in **b**–**e**). Asterisks indicate in **a** and **e** significant differences between the cell lines, in **b**–**d** to zero. Hash signs indicate in **c**–**e** significant differences between the cell lines

**Fig. 3:**
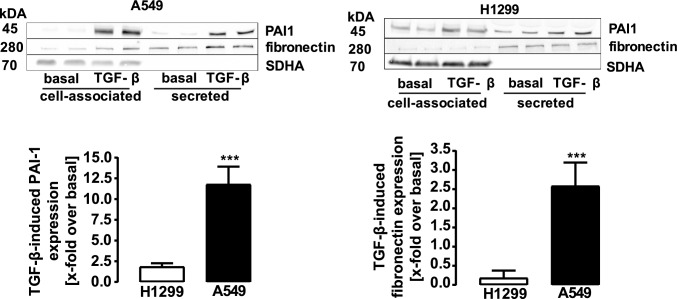
Detection of PAI-1 and fibronectin protein levels in NSCLC cells. Cells were stimulated with TGF-β (2 ng/ml) for 24 h and protein amount of PAI-1, fibronectin or SDHA (loading control) determined. SDHA control of the cellular fraction was also used for the secreted fraction. Statistical analysis was performed by student´s t-test. Asterisks indicate significant differences between the two cell lines

TGF-β is a major regulator of plasmin activity and NSCLC migration (Chen et al. 2019). Its inhibitory actions on plasmin activity are attributed to the activation of SMAD transcription factors and subsequent PAI-1 expression. Thus, we next tested the effects of a 24 h TGF-β-treatment on plasmin activity in NSCLC cell lines. As expected, TGF-β significantly reduced plasmin activity in A549 cells (Fig. 2A, B). In clear contrast, the cytokine dramatically enhanced plasmin activity in H1299 cells. Hence, in the presence of TGF-β, the metastatic-like cell line exhibited the expected elevation in plasmin activity compared to cells derived from a solid tumor. In line with its inhibitory actions on plasmin activity in A549 cells, TGF-β induced dramatic expression of the *SERPINE-1* gene (Fig. 2C), as well as phosphorylation of the SMAD-2 transcription factor (Fig. 2D) and activation of a SMAD-3/4-dependent reporter plasmid (Fig. 2E). This pathway was dramatically reduced on all three levels in H1299 cells (Fig. 2C–E). TGF-β induced PAI-1 protein expression was also strongly pronounced in A549 but barely detectable in H1299 cells (Fig. 3). Fibronectin is a plasmin substrate and a critically important ECM protein (Deryugina and Quigley 2012). When fibronectin expression was analyzed after TGF-β stimulation in the supernatant of NSCLC cells, dramatically increased protein levels were found for A549 but not for H1299 cells (Fig. 3). Hence, we provide conclusive data indicating that the TGF-β/SMAD/PAI-1 pathway is strongly suppressed in H1299 cells, leading to lesser inhibition of plasmin activity compared to A549 cells. However, although these data explain that TGF-β does not decrease plasmin activity in H1299 cells, they cannot account for the observed increase.

**Fig. 4:**
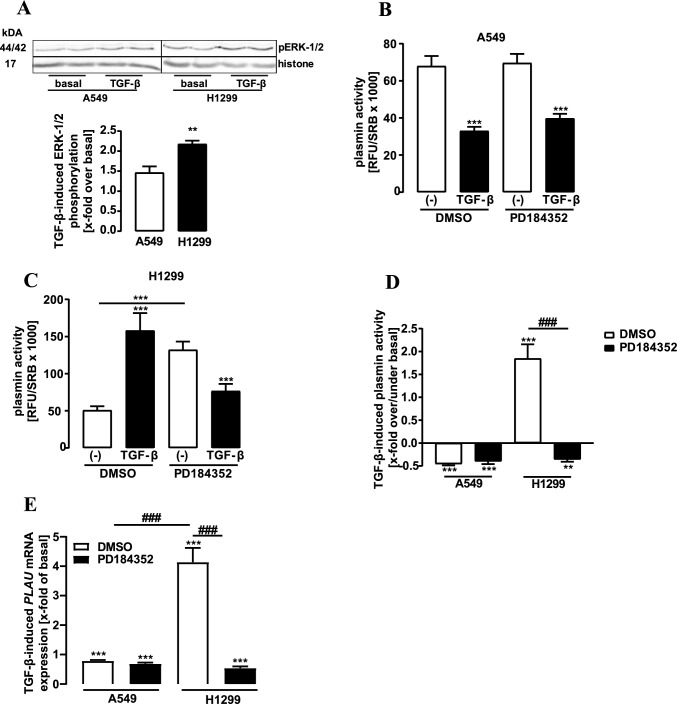
Detection of ERK-1/2 phosphorylation, plasmin activity and mRNA levels in NSCLC cells. **a** Phosphorylation of ERK-1/2 was detected by western-blotting using a p-ERK-1/2 specific antibody in cells stimulated with TGF-β (2 ng/ml) for 24 h. Detection of histone-3 served as a loading control. D-Val-Leu-Lys-AMC (50 µM) was incubated with the supernatant of A549 **b** or H1299 **c** cells stimulated for 24 h with DMSO (0.1%, 24 h), TGF-β (2 ng/ml, 24 h), PD-184352 (10 µM, 24 h) alone or along with TGF-β (2 ng/ml, 24 h) and fluorescence measured. **d** Same data show in **b** and **c** were normalized by setting DMSO or PD-184352 to 100%. **e** mRNA levels of *PLAU* were detected by qRT-PCR in NSCLC cells stimulated for 24 h with DMSO (0.1%, 24 h), TGF-β (2 ng/ml, 24 h), PD-184352 (10 µM, 24 h) alone or along with TGF-β (2 ng/ml, 24 h). Statistical analysis was performed using two‐way ANOVA (**b**–**e**) followed by Tukey´s post-test or one- and two-sample t-test **a** and **d**. Asterisks indicate in **a** significant differences between the cell lines, in **b** and **c** between inhibitor and control, in **d** to zero and in **e** to 1.0. **d**, **e** Hash signs indicate significant differences between the cell lines

We next analyzed effects of TGF-β on ERK-1/2 phosphorylation in H1299 cells (Fig. 4A). We found that TGF-β-induced ERK-1/2 phosphorylation is also higher in H1299 than in A549 cells. Thus, we next detected plasmin activity in both cells in the presence of the ERK-1/2 inhibitor. PD-184352 was without any effects on A549 cells (Fig. 4B). In H1299 cells, the inhibitor enhanced, similar to our data shown in Fig. 1H, basal plasmin activity. Intriguingly, when ERK-1/2 activity was blocked, effects of TGF-β on plasmin activity were reversed, so that the cytokine now inhibited protease activity (Fig. 4C). In fact, when normalized to the basal effects of PD-184352, actions of TGF-β on plasmin activity were indistinguishable in both NSCLC cell lines (Fig. 4D). Increased uPA expression by TGF-β could be a straightforward explanation for increased plasmin activity in H1299 cells. Thus, we analyzed effects of TGF-β on *PLAU* mRNA expression. We found that after stimulation with TGF-β, *PLAU* mRNA levels were dramatically increased in H1299 and slightly decreased in A549 cells (Fig. 4E). Moreover, the ERK-1/2 inhibitor abolished TGF-β-promoted *PLAU* expression in H1299 cells and led to a decrease similar to the one observed in A549 cells. Hence, we provide significant evidence suggesting that TGF-β increased plasmin activity in H1299 cells due to reduced SMAD and enhanced ERK-1/2 activation leading to less PAI-1 but enhanced uPA protein levels.

### Effects of exogenous Plg and serum on plasmin activity in NSCLC cells

**Fig. 5:**
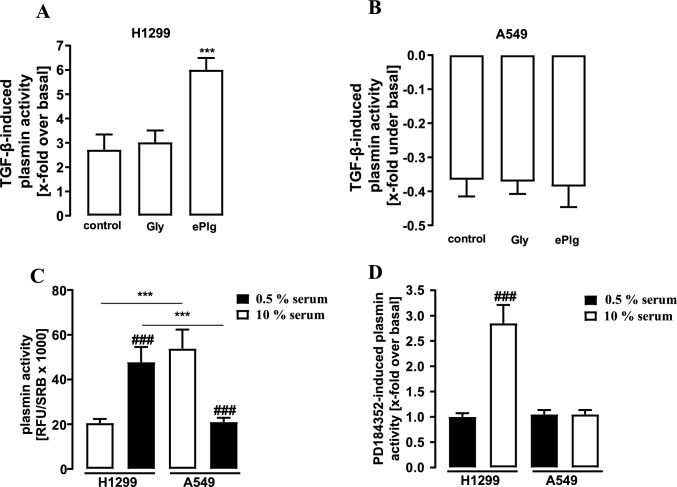
Detection of plasmin activity in NSCLC cells. D-Val-Leu-Lys-AMC (50 µM) was incubated with the supernatant of NSCLC cells and fluorescence measured. **a** H1299 or **b** A549 cells were stimulated for 24 h with glycerin (Gly, 0.1%), or exogenous plasminogen (ePlg, 5 µg/ml) alone or along with TGF-β (2 ng/ml). **c** NSCLC cells were cultured for 24 h with medium containing 10% serum or no serum. **d** NSCLC cells were cultured for 24 h with medium containing 10 or 0.5% serum 24 h and DMSO (0.1%, 24 h) or PD-184352 (10 µM, 24 h). Statistical analysis was performed using two‐way ANOVA followed by Tukey´s post-test. Asterisks indicate in **a**, **b** significant differences between Gly and ePlg, in **c**, **d** between the cell lines. In **c**, **d** hash signs indicated differences between 10 or 0.5% serum

Until this point, we detected plasmin activity without adding exogenous plasminogen (ePlg). Addition of ePlg would only increase signals in the plasmin assay when endogenous Plg levels are not sufficient to saturate uPA. Because TGF-β dramatically enhanced uPA expression in H1299 cells, we postulated that we might even underestimate the possible effects of TGF-β on plasmin activity due to insufficient Plg levels. Indeed, when ePlg was added to H1299 cells an increase in TGF-β-induced plasmin activity was observed from 2.9 ± 0.3 fold over basal to 5.8 ± 0.5 (Fig. 5A). In contrast, ePlg did not alter effects of TGF-β on plasmin activity in A549 cells (Fig. 5B), which is in accordance with the absent uPA induction by the cytokine. So far, we provide evidence that ERK-1/2 activity affects basal and TGF-β-induced plasmin activity in H1299 but not in A549 cells. Because serum is a strong ERK-1/2 activator and we performed all experiments shown so far with 10% of serum, we wondered next, how the serum concentration affects our data. As shown in Fig. 5C, reducing the serum concentration to 0.5% serum for 24 h dramatically increased plasmin activity in H1299 but decreased it in A549 cells. Thus, the metastatic-like phenotype of H1299 cells is also dependent on the culture conditions. In line with this notion, with 0.5% of serum, PD-184352 had no effects on plasmin activity in both cell lines (Fig. 5D).

### Dual role of TGF-β in NSCLC cell migration and death

**Fig. 6:**
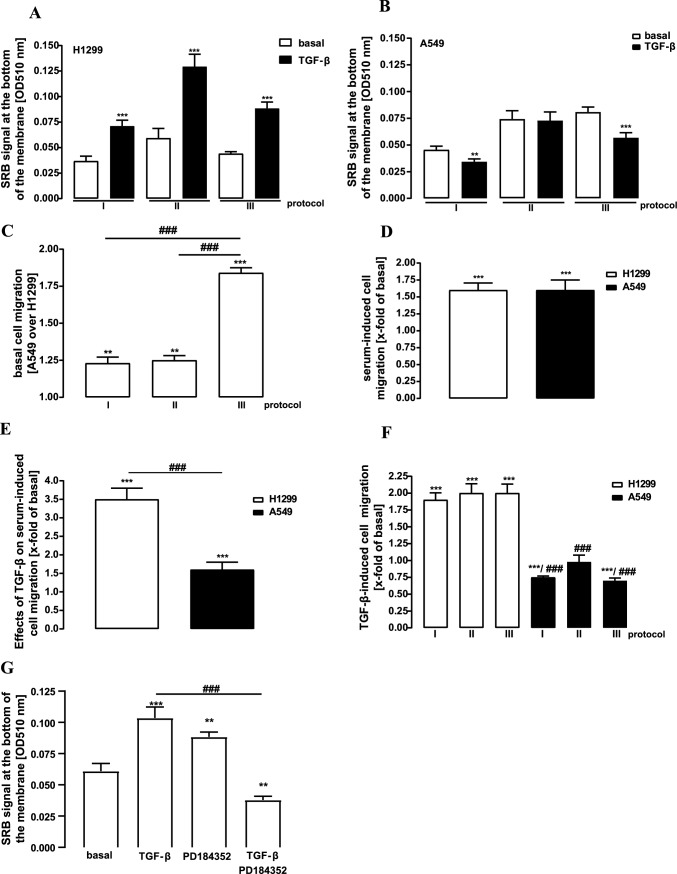
Detection of NSCLC cell migration by boyden chamber migration assays. Cells were seeded on the upper chamber 24 h before the experiment and then stimulated or not by adding TGF-β (2 ng/ml) for 24 h to the upper chamber. Three distinct protocols were applied: I) 0.5% FBS in both chambers, II) 0.5% in the upper and 10% FBS in the lower chamber and III) 10% FBS in both chambers. After 24 h of stimulation, migrated cells at the bottom of the membrane were detected by SRB. In **a**, **b** bars represent SEM of OD_510_ values. **c** basal cell migration of A549 cells was divided by the values of H1299 cells for each protocol and presented as SEM of x-fold. **d** Serum-induced cell migration (basal of protocol II over basal of I) is given as x-fold over basal. **e** Effects of TGF-β on serum-induced cell migration (TGF-β of protocol II over basal of I) is given as x-fold over basal. **f** Effects of TGF-β on cell migration (TGF-β over basal of each protocol) is given as x-fold of basal. **g** Cells were stimulated with TGF-β, PD-184352 or co-stimulated applying protocol III. Statistical analysis was performed using two‐way ANOVA followed by Tukey´s post-test or one-sample t-test. In **a** and **b** asterisks indicate significant differences between TGF-β stimulated cells, in (**c** to **f**) to 1.0. Hash signs indicate significant differences between cell types, in **g** between PD-184352 and control

Distinct effects of serum on plasmin activity in NSCLC cells are of particular interest, when considering cell migration assays. Effects of TGF-β on cell migration were frequently measured when migration was induced by a serum gradient (0.5–10%) (Chen et al. 2019; Wu et al. 2019). Under these conditions, TGF-β-induced migration of A549 cells, despite its inhibitory effects on plasmin activity. Thus, in order to investigate the effects of plasmin activity on NSCLC cell migration under basal conditions and after TGF-β stimulation, we detected cell migration in a boyden chamber assay applying three distinct protocols: I) 0.5% serum in both chambers, II) 0.5% in the upper and 10% serum in the lower chamber and III) 10% serum in both chambers (Fig. 6A, B). We found higher A549 basal cell migration in all protocols, particularly with protocol III (Fig. 6C), which matches the conditions of the plasmin assay (Fig. 2A). Serum-induced cell migration was similar in both cell lines (Fig. 6D). Addition of TGF-β to the upper chamber dramatically enhanced serum-induced H1299 cell migration and to a lesser extend migration of A549 cells (Fig. 6E). Finally, TGF-β induced in all three protocols migration of H1299 cells, but decreased A549 cell migration when protocols with no serum gradient were applied (Fig. 6F). Next, we analyzed the correlation between ERK-1/2 and plasmin activity in H1299 cell migration using protocol III (Fig. 6G). Similar to the enhancing effects of PD-184352 on basal plasmin activity (Fig. 1H), the ERK-1/2 inhibitor induced migration of H1299 cells. Furthermore, when PD-184352 was applied along with TGF-β, it reversed the migratory effects of the cytokine towards anti-migratory actions, which is in clear accordance with the actions of the ERK-1/2 inhibitor on TGF-β-induced plasmin activity shown in Fig. 4C, D.

**Fig. 7:**
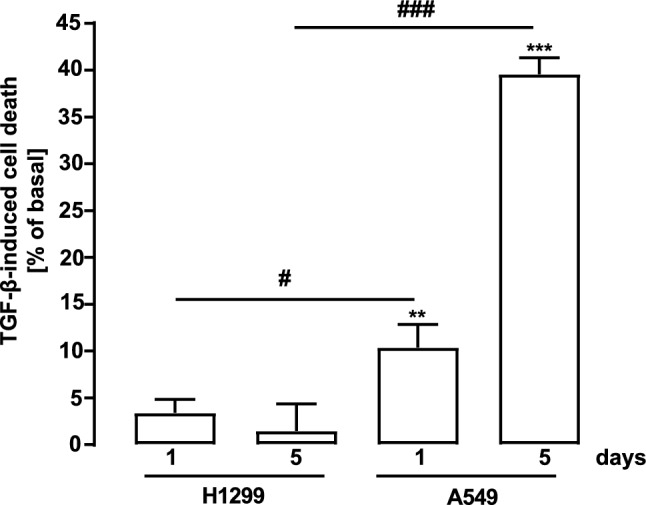
Effects of TGF-β on NSCLC cell proliferation. Cells were seeded in 96-well plates 24 h before the experiment. Cells were then stimulated or not with TGF-β (2 ng/ml) for 1 or 5 d and the amount of cells detected by SRB. Data were normalized by setting unstimulated cells to 100% and cell death calculated as a reduction in percentage. Statistical analysis was performed using two‐way ANOVA followed by Tukey´s post-test or one-sample t-test. In Asterisks indicate significant differences to zero and hash signs between cell types

A paradoxical role in NSCLC has been attributed to TGF-β, because it acts as a tumor suppressor in early-stage tumors, but promotes tumor progression and metastasis in later stages (Zhang et al. 2014). After providing evidence that TGF-β increases both plasmin activity and migration in metastatic H1299 cells, we next analyzed effects of prolonged TGF-β stimulation on toxicity in NSCLC cells (Fig. 7). After 5 days of stimulation, TGF-β reduced the amount of A549 cells by 39 ± 4%, whereas it had no effects on the number of H1299 cells, confirming the anti-proliferative action of TGF-β on early-stage lung tumor cells.

**Fig. 8:**
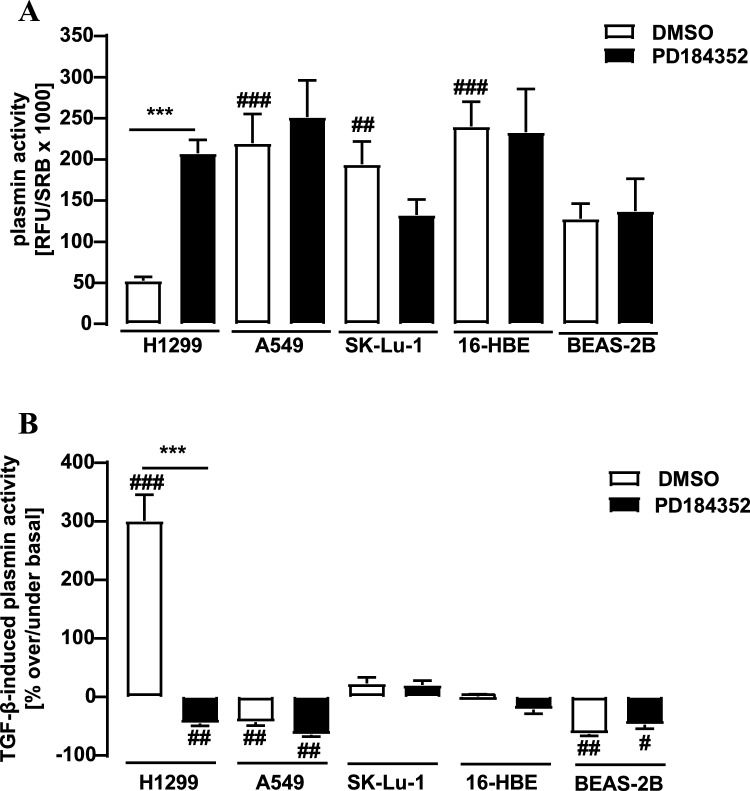
Detection of plasmin activity in lung cells. D-Val-Leu-Lys-AMC (50 µM) was incubated with the supernatant of A549, SK-Lu-1 cells (adenocarcinoma), H1299 cells (large cell carcinoma), 16-HBE or BEAS-2B cells (non-cancer lung epithelial cells) and fluorescence measured. **A** basal plasmin activity was measured after incubation of the cells with PD-184352 (10 µM) or DMSO (0.1%) for 24 h. In **B** Cells were incubated for with PD-184352 (10 µM) or DMSO (0.1%) and with or without TGF-β (2 ng/ml) for 24 h and fluorescence measured. Plasmin activity detected for the control was set to 100%. Statistical analysis was performed using two‐way ANOVA followed by Tukey´s post-test or one-sample t-test. Asterisks indicate significant differences to PD184352. Hash signs indicate significant differences to H1299 cells (DMSO) in **A** and to zero in **B**

Overall, we reveal significant distinctions in the regulation of plasmin activity between A549 adenocarcinoma cells from a primary lung tumor and metastatic H1299 large cell lung carcinoma cells. In order to gather additional data about the uniqueness of H1299 cells, we finally analyzed plasmin activity in two immortalized but non-cancer lung bronchial epithelium cell lines (16-HBE and BEAS-2B) and a second cell line from a primary lung adenocarcinoma (SK-Lu-1). Lowest basal plasmin activity was again observed in H1299 cells and blockade of ERK-1/2 activity exclusively increased plasmin activity in H1299 but not in all other tested cell types (Fig. 8A), highlighting the unique role of ERK-1/2 in plasmin activity of H1299 cells. TGF-β significantly inhibit plasmin activity in BEAS-2B but not in SK-Lu-1 or 16-HBE cells (Fig. 8B), indicating that the enhancing effects of TGF-β on plasmin activity is also unique for H1299 cells. In line with this notion, blockade of ERK-1/2 activity only affected TGF-β-induced plasmin activity in H1299 cells (Fig. 8B).

## Discussion

Advent of NSCLC is a very serious condition. Increased plasmin activity is key in the formation and progression of metastasis. Hence, plasmin activity of metastatic cells is almost directly linked to the early death of human beings and knowledge about the regulation of plasmin activity in metastatic cells holds the valuable chance to identify novel therapeutic targets as well as to develop new strategies against lung cancer.

Here we used two NSCLC cell lines with distinct metastatic potential and compared plasmin activity and cell migration under multiple conditions. We surprisingly found that under standard culture conditions metastatic-like H1299 cells migrated less than A549 cells. These data correlated well with lower plasmin activity due to enhanced PAI-1 expression and ERK-1/2 activity. ERK-1/2 are at the end of the RAS/RAF/MEK pathway, which is a key regulator of cell migration, differentiation and proliferation (Cobb et al. 1994; Hilger et al. 2002). RAF-1 is a proto-oncogene with dramatic implication on many tumors including NSCLC. In fact, high RAF-1 expression in NSCLC further minimizes the 5-year survival rate from 5 to even less than 3% (Tian et al. 2018). However, to the best of our knowledge, the ERK-1/2 pathway has not yet been linked to plasmin activity in NSCLC cells. H1299 cells have been reported to express higher RAF-1 levels than A459 cells and to contain RAS-mutants that are constitutively bound to RAF-1 (Li et al. 2013; Qiu et al. 2019; Tian et al. 2018). Herein, we confirmed high *RAF-1* mRNA levels and found increased ERK-1/2 activity in H1299 cells. The promoter of the *SERPINE-1* gene contains non-overlapping binding sites for SMAD-3/4 and AP-1 transcription factors, consisting of c-FOS and c-JUN proteins (Dennler et al. 1998). It has been shown that H1299 cells overexpress SMAD6 proteins, which are negative regulators of SMAD-3/4 (Jeon et al. 2008). Thus, enhanced SMAD-signaling most likely does no account for enhanced PAI-1 protein levels. In line with these data, we observed significant less SMAD-3/4-dependent reporter activity in H1299 compared to A549 cells. In contrast, we found enhanced ERK-1/2 activity and c-FOS or c-JUN expression. Thus, we favor a model in which increased RAF-1 levels in H1299 enhance PAI-1 expression and dampen plasmin activity via AP-1. This notion is supported by the inhibitory effects of the ERK-1/2 or the AP-1 blocker on *SERPINE-1* mRNA levels and by their enhancing effects on plasmin activity. Hence, we provide first data indicating that the RAS/RAF/MEK pathway inhibits migration of metastatic H1299 cells by lowering plasmin activity via PAI-1. Accordingly, the ERK-1/2 inhibitor forced migration of H1299 cells. Interestingly, this new correlation between the RAF/MEK/ERK-1/2 pathway and plasmin activity was unique for H1299 cells, because the ERK-1/2 inhibitor did not at all affect plasmin activity of A549 cells. Thus, it appears that mutationally enhanced ERK-1/2 activity inhibits plasmin activity in NSCLC. In such a scenario, ERK-1/2 activity may counteract metastatic cell migration. It should be noted that A549 and H1299 cells not only differ in the metastatic potential but also in their lineage, because A549 cell derived from an adenocarcinoma and H1299 from a large cell carcinoma. Thus, additional studies are required to further dissect underlying mechanisms responsible for the distinctions between the two cell lines. However, the comparison with an additional adenocarcinoma and two non-cancer lung cell lines further revealed the unique role of ERK-1/2 activity in the regulation of plasmin activity in H1299 cells.

In the presence of TGF-β, H1299 cells exhibited higher migration and plasmin activity compared to A549 cells. In fact, when five distinct lung cell lines were compared, H1299 cells were the only cell system with enhanced plasmin activity after TGF-β stimulation. These data clearly highlight the role of TGF-β as a mediator of NSCLC-large cell carcinoma metastasis. We found that this role correlates with a shift of the PAI-1/uPA expression ratio to the side of PAI-1 in A549 cells and towards uPA in H1299 cells. Of note, inhibition of ERK-1/2 activity completely reversed the effects of TGF-β on H1299 cells and induced an A549-like phenotype. Hence, our study provides new insights into the cellular events induced by TGF-β and responsible for the metastatic-like phenotype of NSCLC cells. TGF-β acts as a tumor suppressor in early-stage tumors, but promotes tumor progression and metastasis in later stages (Zhang et al. 2014). Here we confirmed that TGF-β inhibits A549 but not H1299 cell proliferation. Interestingly, it appears that TGF-β levels increase during NSCLC progression and that high TGF-β levels even aggravate the prognosis (Huang et al. 2014). Herein, we offer a cellular explanation for this bad prognosis. TGF-β induces signaling in NSCLC that promotes migration (rho/rac-mediated cytoskeleton remodeling) or weakens migration (SMAD-dependent PAI-1 expression). Metastatic H1299 cells have been shown to overexpress inhibitory SMAD-6 proteins, thus it has been postulated that the SMAD pathway is weakened in these cells (Jeon et al. 2008). In line with these data, we observed reduced TGF-β-promoted *SERPINE-1* induction and PAI-1 protein levels. Reduced TGF-β-promoted PAI-1 expression should allow rho/rac to influence migration even further. Indeed, we observed much stronger TGF-β-induced H1299 than A549 cell migration under all tested conditions. Noteworthy, this effect is not only caused by reduced *SERPINE-1* induction. We observed that TGF-β strongly induced uPA expression in H1299 cells. Hence, in the metastatic NSCLC cell line, TGF-β shifts the PAI-1/uPA expression ratio decisively towards uPA and thus enhances two cell migration promoting pathways: cytoskeleton remodeling and plasmin activity. From the perspective of a patient, this is a devastating scenario. However, this scenario could be fully reversed by the addition of an inhibitor of the RAS/RAF/MEK pathway. RAF and MEK inhibitors such as selumetinib and trametinib have been tested as a potential treatment of NSCLC (Han et al. 2021). Interestingly, at early stages of NSCLC, monotherapy with MEK inhibitors is only weakly effective. In line with these data, we observed beneficial effects of a MEK inhibitor on cell migration or plasmin activity only in H1299 cells and in the presence of TGF-β. A combination of MEK and RAF inhibitors demonstrated improved efficacy in later stages of NSCL. In fact, trametinib combined with dabrafenib, is a FDA and EMA approved therapy for NSCLC patients with RAF mutants (Planchard et al. 2016a, b; Spain et al. 2016). Noteworthy, these inhibitors were applied in order to block the RAS/RAF/MEK pathway in the context of epidermal growth factor receptor mediated activation. Data presented herein suggest that blocking the RAS/RAF/MEK pathway in the context of TGF-β-mediated activation by TGF-β signaling specific antagonists such as galunisertib, might be an even better approach.

## Conclusion

Our study reveals an exclusive role for ERK-1/2 in the regulation of plasmin activity and migration of metastatic NSCLC-H1299 cells. ERK-1/2 dampens basal plasmin activity and migration but enhances the effects of TGF-β on both. Thus, blocking TGF-β-promoted ERK-1/2 activity might be a straightforward approach to inhibit NSCLC metastasis, in particular in patients with high RAF-1 and TGF-β levels.

## Data Availability

All the data that support the findings of this study are available from the corresponding author upon reasonable request.

## Abbreviations

AP-1: Activator protein-1
BM: Basement membrane
D-Val-Leu-Lys-AMC: D-Val-Leu-Lys-7-amido-4-methylcoumarin
ECM: Extracellular matrix
ERK-1/2: Extracellular-regulated kinases-1/2
NSCLC: Non-small cell lung carcinoma
Plg: Plasminogen
uPA: Urokinase plasminogen activator (protein)
PAI-1: Plasminogen activator inhibitor-1 (protein)
PLAU: Plasminogen activator, urokinase (gene)
SERPINE1: Serpin family E member 1 (gene)
SDHA: Succinate dehydrogenase
SRB: Sulforhodamine B
TGF-β1: Transforming growth factor-β1
